# Low fat intake is associated with pathological manifestations and poor recovery in patients with hepatocellular carcinoma

**DOI:** 10.1186/1475-2891-12-79

**Published:** 2013-06-08

**Authors:** Kazuki Yamada, Takeshi Suda, Yuko S Komoro, Tsutomu Kanefuji, Tomoyuki Kubota, Toshiko Murayama, Hideaki Nakayama, Yutaka Aoyagi

**Affiliations:** 1 Division of Gastroenterology and Hepatology, Graduate School of Medical and Dental Sciences, Niigata University, Niigata 951-8122, Japan; 2 Nutrition Control Center, Niigata University Medical and Dental Hospital, Niigata 951-8520, Japan; 3 Division of Pneumology, Niigata University Medical and Dental Hospital, Niigata 951-8520, Japan

**Keywords:** Hepatocellular carcinoma, Protein-energy malnutrition, Minimal hepatic encephalopathy, Non-protein respiratory quotient

## Abstract

**Background:**

This study aimed to clarify whether dietary deviation is associated with pathological manifestations in hepatocellular carcinoma (HCC) patients.

**Methods:**

Dietary intake was estimated in 35 HCC cases before and after hospitalization by referencing digital camera images of each meal. Pathological conditions were evaluated in nitrogen balance, non-protein respiratory quotient (npRQ), neuropsychiatric testing and recovery speed from HCC treatment.

**Results:**

On admission, nitrogen balance and npRQ were negative and less than 0.85, respectively. Five patients were judged to have suffered from minimal hepatic encephalopathy that tended to be associated with a lowered value of npRQ (p = 0.082). The energy from fat intake showed a tendency of positive correlation with npRQ (p = 0.11), and the patients with minimal hepatic encephalopathy took significantly fewer energy from fat (p = 0.024). The energy difference from fat between diets at home versus those in the hospital showed a significant positive correlation with npRQ change after admission (p = 0.014). The recovery speed from invasive treatments for HCC showed a significant negative correlation with npRQ alteration after admission (p = 0.0002, r = −0.73).

**Conclusions:**

These results suggest the lower fat intake leads to deterioration of energy state in HCC patients, which associates with poor recovery from invasive treatments and various pathological manifestations.

## Background

Hepatocellular carcinoma (HCC) is the third most common cause of cancer death in the world and accounts for over 500,000 deaths a year [1]. HCC is a unique type of cancer, which mostly arises in livers with chronic necroinflammatory damage due to various etiologies such as hepatitis C virus infection and non-alcoholic steatohepatitis [2]. Because long lasting necroinflammation leads to reductions of functional liver reserves, the patient’s survival cannot be predicted from cancer stage alone, which is done in the other types of cancer. It has been reported that a system integrating both cancer stage and functional liver reserve can accurately stratify patient survival rates [3]. For example, the Japan integrating scoring indicates that the functional liver reserve as assessed by the Child-Pugh scoring system has an impact on a patients’ survival equivalent to anatomical cancer extension [4]. Thus, it is advisable to manage functional liver reserves in HCC patients in parallel with their cancer treatment in order to improve survival.

In terms of nutritional state, a characteristic feature of patients suffering from liver cirrhosis is protein-energy malnutrition (PEM) [5,6]. An insufficient energy intake of less than 30 kcal/kg has been reported to be associated with a poor prognosis in cases of liver cirrhosis [7]. Randomized prospective case control studies have revealed that nutritional intervention in order to support sufficient energy intake significantly improves patient survival [8-10]. Unfortunately, it is common for cirrhotic patients to present with comorbidities such as hypermetabolism, inefficient digestion and anorexia [11], which counteract the beneficial effects of sufficient energy intake. The occurrence of hepatic encephalopathy due to an improper protein diet makes it even more difficult to practically maintain nutritional-energy balance during cirrhosis.

Along with energy intake, the body aims to maintain energy balance adopting various ways. In patients with anorexia nervosa, the physiological adaptation to malnutrition is expressed as the refeeding syndrome, when the extra energy is administered even with an appropriate amount to body size [12,13]. A daily consumption of a high-fat diet alters the homeostatic regulation [14-16]. Lean people consuming a high-fat diet are associated with increased energy expenditure at rest and a relatively higher fat oxidation to avoid weight gain [17]. These facts suggest that a nutritional intervention should be adjusted not statically but dynamically in association with personal daily life. In this report, we evaluated dietary intake both at home and in hospital among patients with HCC from the points of PEM, minimal hepatic encephalopathy (MHE) and recovery from cancer treatment, and show that dietary deviation is an important consideration when invasive treatments are planned. In addition, the impact of nutritional intervention is discussed for the optimal management of HCC.

## Methods

### Patients

Thirty-five consecutive cases suffering from HCC with various histories of liver disease were enrolled in this study (Table 1, Group 1). When hospital admission was primarily to treat HCC, a digital camera and questionnaires were provided to record the diet at home several days before admission. A computer-aided neuropsychiatric test (NP test) and assessment of body composition based on a bioelectrical impedance analysis using InBody system (BIOSPACE, Tokyo, Japan) were performed upon admission. In addition, a dietician calculated energy intake based on Japanese dietary allowance according to home photo images, which were obtained at least three consecutive days prior to the admission both before and after each meal including snacks, and descriptions from the questionnaires. In patients without any special comorbidity such as diabetes mellitus, a regular hospital diet of 1800 kcal/day was served. Any nutritional support including branched-chain amino acids (BCAA) was kept as it was in the outpatient clinic. On the next day, day 1, nitrogen balance and non-protein respiratory quotient (npRQ) were evaluated. On day 4 after admission, one day before the application of invasive therapy, the NP test, nitrogen balance and npRQ were assessed again. The questionnaires and recording of digital photos before and after each meal were continued until day 4 in order to calculate actual energy intake for the hospital and non-hospital provided diet. This study was conducted according to the guidelines laid down in the Declaration of Helsinki and all procedures involving human subjects/patients were approved by the Niigata University Graduate School of Medical and Dental Sciences Human Research Committee. Written informed consent was obtained from all patients for publication of individual clinical details.

**Table 1:** **Summary of patients**’ **characteristics**

**Items**	**Group 1 (*n*** **=** ***35*)**	**Group 2 (*n*** **=** ***20*)**
Age	68.5 ± 8.2	71.0 ± 8.2
Gender (male/female)	28/7	14/6
Body mass index	24.3 ± 3.2	24.6 ± 3.5
Background (HBV/HCV/Alcohol/NASH/PBC/AIH)	12/14/6/3/1/0	4/11/3/1/0/1
Child-Pugh score (5/6/7/8/9)	20/7/6/0/2	11/5/3/0/1
TNM stage (I/II/III/IV)	18/9/3/5	6/5/4/5
Treatment (RFA/TACE/TOCE/HAIC/Sorafenib/No)	8/6/3/11/1/6	4/6/3/7/0/0
BCAA supplementation (+/−)	21/14	11/9
PT-INR on admission	1.12 ± 0.09	1.09 ± 0.08

*HBV* hepatitis B virus, *HCV* hepatitis C virus, *NASH* nonalcoholic steatohepatitis, *PBC* primary biliary cirrhosis, *AIH* autoimmune hepatitis, *TNM* tumor-node-metastasis, *RFA* radiofrequency ablation, *TACE* transcatheter hepatic arterial chemoembolization, *TOCE* transcatheter oily chemoembolization, *HAIC* hepatic arterial infusion chemotherapy, *BCAA* branched-chain amino acids, *PT*-*INR* prothrombin time-international normalized ratio.

### Neuropsychiatric test

When a patient received an abnormal value for the revised version of the Hasegawa dementia scale [18], he/she was excluded from this study. MHE was evaluated using a computer-aided quantitative NP test [19], which consisted of the eight following categorical tests: number connection tests A and B, a figure position test, a digit symbol test, a block design test, and reaction time tests A, B and C. Because the test results were affected by age, abnormality in each category was originally defined as values beyond the 90th percentile for an age-matched value over a 5-year interval, which was obtained from 542 healthy Japanese volunteers from 40 to 69-years old. Unfortunately, there were many patients who were 70-years old or older. To make an assessment for a patient over 69-years old, a linear regression curve was deduced that excluded reaction time tests, which were fit by second-order polynomial non-linear regression. Each regression analysis showed a significant correlation giving r squares of 0.95, 0.93, 0.77, 0.99, 0.91, 0.94, 0.93 and 0.82, respectively. Thus, in this study, value for each test was decided to be abnormal when it exceeded the 90th percentile, which was calculated from the regression curve for the same age. Because many elderly patients do not react properly to the test due to a lack of experience using a computer, MHE was not diagnosed if the abnormal value appeared in one category but normal during the second evaluation. Instead, MHE was diagnosed when an abnormal value was reproducibly recorded even in one category or when abnormal values were obtained at least once in multiple categories.

### Measurements of non-protein respiratory quotient and estimations of energy metabolism

Energy metabolism was analyzed using an indirect calorimeter, AERO MONITOR AE300S (Minato Medical Science Co., Ltd. Osaka, Japan), on day 1 and day 4 after overnight bed rest and fasting. Every five seconds, oxygen consumption (VO2) and carbon dioxide production (VCO2) were measured until steady-state values were obtained over two consecutive minutes. A steady state was defined by a variation between 5% and 10% in the average value for oxygen consumption and carbon dioxide production over 3 minutes. npRQ was calculated as an average of the following ratio: VCO2/VO2. Urine urea nitrogen (UUN) was measured by urease indophenol method in urine that was collected throughout the day without any intravenous infusion. Resting energy expenditure (REE) was calculated according to the following formula [20]: ((15.913 × VO2 + 5.207 × VCO2) × 1.44 - 4.646 × TUN) × 0.239. TUN (total urea nitrogen) was calculated in the urine, which was collected over 24 hours, as UUN + 4 if UUN exceeded 15 g/day; otherwise, TUN was calculated as UUN × 1.17 + 0.7. Basal energy expenditure (BEE) was estimated using a Harris-Benedict equation, in which BEE was 66.5 + (13.75 × body weight in kg) + (5.003 × body height in cm) - (6.775 × age) for males and was 655.1 + (9.563 × body weight in kg) + (1.850 × body height in cm) - (4.676 × age) for females. The daily energy requirement was estimated from REE or BEE by multiplying by stress and activity coefficients of 1.1 and 1.3, respectively. The stress coefficient was selected between no stress; 1.0 and suffering from advanced cancer; 1.2, while the activity coefficient was decided between simple walking; 1.2 and light labour; 1.4, respectively. The protein and energy malnutrition was diagnosed in the case that npRQ was less than 0.85 [21] as well as nitrogen balance was negative.

### Normalization of therapeutic invasiveness using prothrombin time

In order to standardize a recovery speed form HCC treatments based on therapeutic intensity, a reduction rate of prothrombin time (PT-INR) was used as an indicator for the intensity. Given nadir as a day showing minimum value of PT-INR after finishing an entire series of treatment against HCC in one admission, a reduction rate of PT-INR was defined as a reduction percentage of PT-INR at nadir against PT-INR on admission. The formula calculating PT-INR reduction rate is as follows where PT-INRad and PT-INRnad indicate PT-INR on admission and at the nadir after a series of treatment, respectively.

PT-INR reduction rate (%) = (PT-INRad – PT-INRnad)/PT-INRad × 100

Then, a recovery speed from treatments for HCC was evaluated based on a length of hospital satay (day) after nadir that was normalized by dividing with the PT-INR reduction rate as follows.

Recovery speed = hospital stay after PT-INRnad/PT-INR reduction rate × 100

### Statistical analysis

Categorical data were compared between two groups using paired or unpaired t tests when groups were matched or unmatched, respectively. Correlation between npRQ and various factors were analyzed by calculating the Pearson correlation coefficient. All analyses were performed using GraphPad Prism 6 software (GraphPad Software, Inc. La Jolla, USA) except for a multivariate linear regression analysis, for which PASW statics 17.0 (SPSS Inc., Chicago, USA) was used, and a two-sided P-value less than 0.05 was considered statistically significant.

## Results

### Patients hospitalized for hepatocellular carcinoma treatments frequently suffered from protein-energy malnutrition

**Figure 1:**
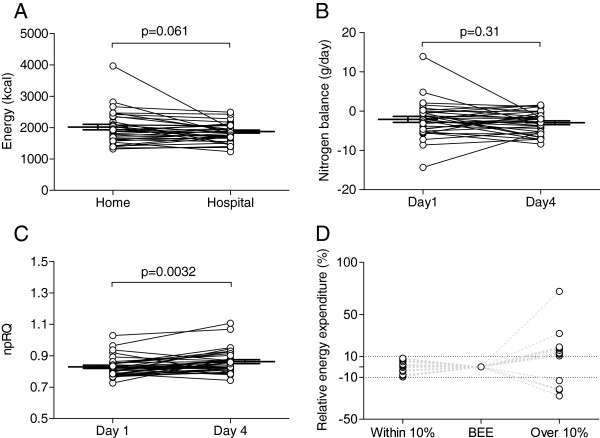
**Energy intake and protein**-**energy malnutrition in patients with hepatocellular carcinoma.** (**A**) The average energy intake of 35 HCC patients at home was 1977 ± 513 kcal/day (mean ± SD) and tended to be higher than the 1834 ± 290 kcal/day in the hospital (p = 0.061). (**B**) Nitrogen balance was measured from a 24 hr specimen of urine and was −2.1 ± 4.5 g/dl and −3.0 ± 2.8 g/dl on days 1 and 4 after admission, respectively. There was no significant difference between these values (p = 0.31). (**C**) Non-protein respiratory quotient on day 1 was 0.83 ± 0.061 and was significantly lower than 0.86 ± 0.075 on day 4 (p = 0.0032). (**D**) Daily energy requirement was calculated based on resting energy expenditure (REE) or the basal energy expenditure (BEE) estimated from the Harris-Benedict equation. When stress and activity indices were set at 1.1 and 1.3, respectively, 15 out of 34 cases showed more than 10% difference between REE-based and BEE-based estimations.

Patients who were admitted for active treatment of HCC had a home diet with an average of 1977 ± 513 (mean ± SD) kcal/day leading to a negative nitrogen balance of −2.1 ± 4.5 g/dl (Figure 1A and 1B). Because among 35 cases, a case could not properly collect urine sample for a day on admission causing loss of UUN data, nitrogen balance and REE were evaluated for 34 cases. The energy malnutrition was also observed in the average npRQ value of 0.83 ± 0.061 on day 1, which was estimated to reflect the energy status at home (Figure 1C). npRQ on day 1 was not significantly correlated with BMI of 24.3 ± 3.2 kg/m^2^ (p = 0.35) nor other representative body composition markers such as intra and extra cellular water, percent body fat or soft lean mass (data not shown). The average energy intake in the hospital was 1834 ± 290 kcal/day and tended to be lower than that at home (p = 0.061, Figure 1A). However, the negative nitrogen balance of −3.0 ± 2.8 g/dl was not significantly different (p = 0.31, Figure 1B), and the energy state was even significantly improved to a normal range of npRQ on day 4 at 0.86 ± 0.075 (p = 0.0032, Figure 1C). The energy requirements were calculated by multiplying 1.1 and 1.3 as stress and activity coefficients, respectively, to REE or BEE (Materials & Methods), which resulted into 1940 ± 385 kcal or 1860 ± 281 kcal/day, respectively. Fifteen out of 34 cases showed more than 10% difference between REE-based and BEE-based estimations (Figure 1D). Although protein-energy malnutrition was observed on day 1, there was no significant difference between total energy intake at home, 1977 ± 513 kcal/day, and the energy requirement calculated from REE, 1940 ± 385 kcal/day, (p = 0.60). There were 21 cases that received the nutritional support of BCAA in 35 cases (Table 1, Group 1), and no significant difference was observed between cases with and without BCAA supplementation in nitrogen balance or npRQ day 1 (p = 0.99 and p = 0.53, respectively).

### Minimal hepatic encephalopathy is associated with energy malnutrition

**Figure 2:**
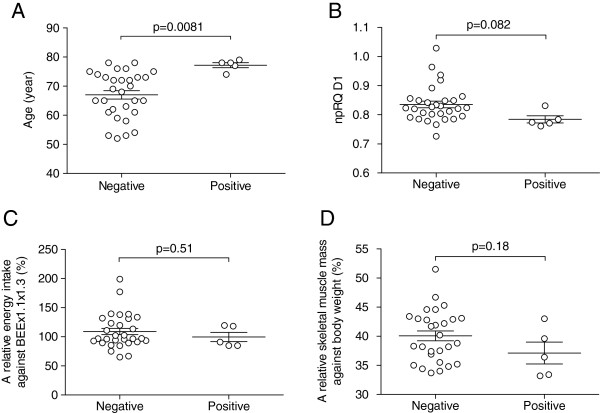
**Relationship between minimal hepatic encephalopathy** (**MHE**) **and various clinicopathological factors.** (**A**) Five patients who were diagnosed with MHE using a computer-aided neuropsychiatric test were significantly older in age (77.2 ± 1.9) than the MHE-negative cases (67.0 ± 8.0) (p = 0.0081). (**B**) npRQ on day 1 in MHE-positive and MHE-negative cases were 0.78 ± 0.027 and 0.84 ± 0.062, respectively, and tended to be lower in MHE-positive cases (p = 0.082). (**C**) Relative energy intakes at home expressed as a percentage to basal energy expenditure multiplied by stress and activity indices of 1.1 and 1.3, respectively, were 109.0 ± 30.0% and 99.7 ± 17.7% in MHE-negative and -positive cases, and were not significantly different each other (p = 0.51). (**D**) Similarly, body compositions of skeletal muscle mass relative to body weight were 40.1 ± 4.5% and 37.1 ± 4.2% in MHE-negative and -positive cases, and were not significantly different each other (p = 0.18).

MHE was diagnosed in 5 cases on the basis of the computer-aided NP test. MHE-positive cases consisted of elderly patients with an average age of 77.2 ± 1.9 years, which was significantly older than the 67.0 ± 8.0 years for the cases that exhibited a negative NP test (p = 0.0081, Figure 2A). The energy state in MHE-positive cases was critically impaired on admission, which was indicated by the npRQ day 1 of 0.78 ± 0.027. These patients tended to be malnourished in comparison to MHE-negative cases with an npRQ of 0.84 ± 0.062 (Figure 2B, p = 0.082). In contrast, there were no significant differences between MHE-positive and -negative groups in regard to energy intake and body composition of skeletal muscle amount as shown in Figure 2C and 2D, respectively (p = 0.51 and p = 0.18, respectively).

### Poor energy intake from fat is associated with minimal hepatic encephalopathy and starvation in patients with protein-energy malnutrition

**Figure 3:**
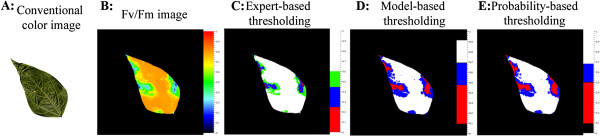
**Effects of energy intake from fat on non**-**protein respiratory quotient and minimal hepatic encephalopathy.** (**A**) There was a tendency of correlation between energy intake from fat relative to total energy intake at home and non-protein respiratory quotient (npRQ) day 1 (p = 0.11, r = 0.28). (**B**) The difference in energy intake from fat between at home and in hospital diets was significantly correlated with the alteration of npRQ day 1 and day 4 (p = 0.014, r = 0.41). (**C**) and (**D**). The relative energy intake from fat to total energy intake was 18.9 ± 3.8% in minimal hepatic encephalopathy (MHE)-positive cases and was significantly lower than 23.6 ± 4.2% in MHE-negative cases (p = 0.024). Even for the cases that were limited to 70-years old or older people, the energy intake from fat was 24.0 ± 4.6% in MHE-negative cases, which was significantly higher than MHE-positive cases (p = 0.040).

The deviation of diet in relation to energy malnutrition and MHE was observed as a reduced energy intake from fat. The relative energy intake from fat at home tended to be correlated with npRQ day 1 as shown in Figure 3A (p = 0.11, r = 0.28). Moreover, the difference in energy from fat between home and hospital diets showed a significant positive correlation with npRQ change between day 1 and day 4 (p = 0.014, r = 0.41, Figure 3B).

In accordance with the relationship between MHE and npRQ as described above, MHE-positive cases relied on significantly fewer energy from fat at home with 18.9 ± 3.8% in comparison to 23.6 ± 4.2% in MHE-negative cases (p = 0.024, Figure 3C). Because MHE-positive cases consisted of significantly older people (70-years old or older) as shown in Figure 2A, the lower intake of energy from fat in MHE-positive cases may be confounded by age differences between MHE-positive and MHE–negative cases. However, given that these patients were 70-years old or older, there was still a significant difference in terms of energy intake from fat. Specifically, an average energy intake from fat of 24.0 ± 4.6% for 14 MHE-negative cases was significantly higher than that for 5 MHE-positive cases (p = 0.040, Figure 3D). Moreover, the difference of energy intake from fat was compared between general population and our cohort using 2010 national surveillance data from Japan. In people with age of 70 or older, there was no significant difference of energy percentage from fat between MHE-negative patients in this study and general population (22.9 ± 10.5% (n = 562), p = 0.69). There was a significant correlation of percent body fat neither with total energy intake nor relative energy intake from fat (p = 0.28 or p = 0.43, respectively).

### Deterioration of npRQ over hospitalization is associated with poor recovery from invasive therapies for hepatocellular carcinoma

The effects of transition from home to hospital diets on energy malnutrition and recovery from invasive therapies were evaluated. Because these patients were treated using various therapeutic options such as transcatheter hepatic arterial chemoembolization or radiofrequency ablation, the invasiveness affected each case differently. To compare the recovery speed among cases, the invasiveness has to be standardized. For this purpose, we employed PT-INR reduction rate (Materials and Methods).

If no treatment was conducted or PT-INR was not reduced after treatments, those cases were excluded from the analysis in order to specifically examine the deterioration-recovery sequence. Furthermore, cases that showed the minimal PT-INR value amid a multiple treatment course were also excluded. Because only 11 cases were qualified for a further analysis from the original 35 cases, additional 9 cases were subjected to analysis of the PT-INR reduction rate and npRQ day 1 and day 4. Finally, a total of 20 cases (Table 1, Group 2) were subjected to further analyses. After these therapies, PT-INR was reduced 5.8 ± 3.8% on its nadir of 5.8 ± 5.1 days after each treatment.

**Figure 4:**
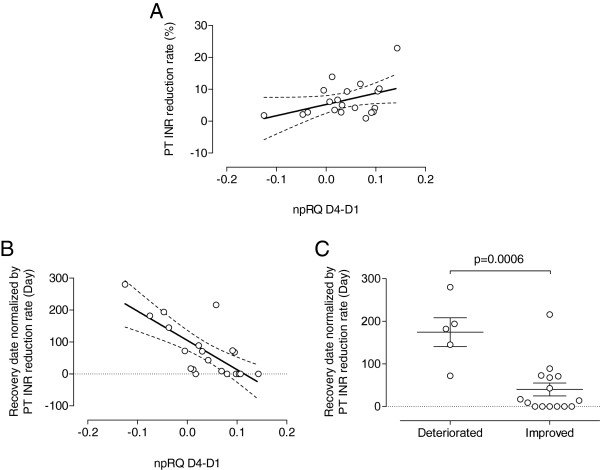
**Association of recovery speed from invasive treatment and non**-**protein respiratory quotient.** (**A**) Prothrombin time (PT-INR) reduction rate, which was the maximal reduction of PT-INR after treatment against the level at admission, was estimated as one of indicators for the magnitude of invasiveness and was not correlated with the non-protein respiratory quotient (npRQ) difference between days 1 and 4 (p = 0.065, r = 0.42). (**B**) The length of hospital stay after the nadir of PT-INR due to a treatment exhibited a significant negative correlation with the npRQ difference between days 1 and 4 after normalization by PT-INR reduction rate (p = 0.0002, r = −0.73). (**C**) The length of hospital stay after the nadir of PT-INR was 175 ± 76 days and 40 ± 59 days in npRQ-deteriorated and npRQ-improved cases after admission, respectively, and significantly different each other (p = 0.0006).

There was no significant correlation between the PT-INR reduction rate and the difference in npRQ between day 1 and day 4 (p = 0.065, r = 0.42, Figure 4A), thereby suggesting that therapeutic option was not selected on the basis of the change in energy state after hospitalization. On the other hand, when the length of hospital stay after the nadir of PT-INR was normalized with the PT-INR reduction rate and used as an indicator of the recovery speed after invasive treatments, the recovery speed showed a significant negative correlation with the difference in npRQ between day 1 and day 4 (p = 0.0002, r = −0.73, Figure 4B). Consistently, the length of hospital stay after the nadir of PT-INR was significantly longer in 5 cases with deteriorated npRQ after admission comparing with that in 15 cases, in which npRQ was improved after admission (175 ± 76 days *vs* 40 ± 59 days, respectively, p = 0.0006, Figure 4C).

In order to confirm that npRQ difference between day 1 and day 4 is a significant determinant for the length of hospital stay after treatment of HCC, a multiple linear regression analysis was performed by employing the recovery speed as a dependent variable. Independent variables were consisted of 8 factors; age, gender, background liver diseases, TNM stage, Child-Pugh score, body mass index, BCAA supplementation, and npRQ difference. In the results, npRQ difference was selected as an only significant explanatory (p = 0.001, Table 2).

**Table 2:** Result of multivariate linear regression analysis for recovery speed

**Variables**	**Unstandardized coefficients**	**Significance**
(Constant)	−27.102	0.908
Age	1.612	0.557
Gender	−33.052	0.368
Background liver disease	−34.672	0.223
TNM Stage	6.114	0.663
Child-Pugh score	−23.669	0.192
Body mass index	7.926	0.174
BCAA supplementation	46.270	0.194
npRQ Day4 - Day1	−1161.490	0.001

*TNM* tumor-node-metastasis, *BCAA* branched-chain amino acids, *npRQ* non-protein respiratory quotient, Day1, the first morning after admission, Day4, the forth morning after admission.

## Discussion

Several lines of evidence strongly indicate that a functional hepatic reserve possesses similar impacts on the survival of HCC cases with anatomical cancer extension [3,4,22-24]. On the other hand, several randomized prospective case control studies have revealed that active nutritional intervention significantly improves the prognosis in patients with liver cirrhosis [8-10]. Taken together with the evidence that energy intake lower than 30 kcal/kg leads to a poor prognosis in cirrhotic patients [7], it is reasonable to assume that a strategy to preserve functional hepatic reserves should be incorporated into a treatment scheme for HCC. In this report, we first evaluated protein-energy status in patients who were facing active interventional treatments for HCC. In these cases, PEM was clearly present on admission as a negative nitrogen balance and an npRQ less than 0.85 in association with MHE that was diagnosed in 5 out of 35 cases. These results strongly suggest that nutritional intervention should be started before hospitalization in patients with HCC. In terms of nutritional support, BCAA supplementation was reported to elongate event-free survival by improving PEM in cirrhotic patients [25], while its efficacy was equivocal in cases receiving radiofrequency ablation as the form of HCC treatment [26]. Although the nitrogen balance and npRQ were not significantly different between cases with and without BCAA supplementation in this study, the limited number of cases does not provide a conclusive result. The significance of BCAA supplementation under active treatments of HCC should be further evaluated in a larger cohort.

It was reported that BEE underestimates energy requirements in patients with liver cirrhosis, which leads to a hypermetabolic state [27]. Consistently, the BEE-based energy requirement calculated from the Harris-Benedict equation was different from the REE-based estimation more than 10% in more than 44% cases in this study. On the other hand, the total energy intake at home was not significantly different from the daily energy requirement, which was estimated from REE by multiplying 1.1 and 1.3 as the stress and activity coefficients, respectively. Although energy equivalents are considered in the REE-based calculation, these PEM suffering patients suggest that liver cirrhosis affected energy state not only by inducing hypermetabolism but also by hampering the absorption and/or efficient usage of nutrients. Furthermore, the increase of npRQ above 0.85 after admission even with consumption of less energy suggests that it is practically difficult to select appropriate activity and stress coefficients. Taken together, it is strongly recommended that the protein-energy state should be used to define an appropriate daily energy intake in cirrhotic patients using indicators such as npRQ.

The computer-aided NP test is one of the few quantitative approaches for the diagnosis of MHE that were recommended in the guidelines provided by the World Congress of Gastroenterology-commissioned Working Party [28] due to their high specificity for diagnosing hepatic encephalopathy [29]. Currently, however, the diagnosis and clinical significance of MHE have not been well defined [30]. While abnormal values at least in two tests among eight subsets were reported to be required achieving 80% of sensitivity [31], the same setting were also employed for diagnostic criteria in NP test consisting of four subsets instead of eight [32]. Another problem for NP test is age dependency [19]. There are no available control data for patients over the age of 69. Hence, it may be difficult to distinguish an early stage of dementia from MHE. In addition, there is a concern that these results may be affected by unfamiliarity with using a computer device, especially when elderly patients are the subjects. In this study, the 90th percentile for normal controls was estimated in each test from a regression line deduced from the values of controls between ages 40 to 69. A high Pearson’s coefficient value for this regression line demonstrated the goodness of fit for all eight categories. Patients who were diagnosed with dementia were excluded from the study on the basis of a revised Hasegawa dementia scale [18]. Although senile decay in reaction time and/or cognition may not be completely excluded from our MHE diagnostic criteria, the lower values of npRQ in all 5 MHE-positive patients strongly suggested that MHE diagnosed by our criteria was associated with functional hepatic reserve. Easy and reliable diagnostic criteria for MHE should be further explored through extensive studies using a larger cohort to prove the clinical significance of MHE in association with the energy malnutrition.

This study suggested that an insufficient fat source impaired the recovery from invasive treatments for HCC in cirrhotic patients. An indicator of energy state, npRQ, was significantly changed after admission in association with the energy difference for fat consumed between home and the hospital. Consistent with the association between MHE cases and lower npRQ, the relative energy from fat was significantly lower in the cases that were diagnosed with MHE. Taken together, it is suggested that energy state should be improved before invasive treatments to promote a rapid recovery, and specifically, energy from fat should be provided at a dose recommended for the regular dietary allowance, which is between 20% to 30% of total energy intake [33,34]. In terms of normalization of therapeutic invasiveness, PT-INR was employed in this study. Although serum concentrations of NH_3_ and total bilirubin were tested for this purpose, these values were prerequisitely altered due to extrahepatic circumstances such as constipation or constitutive jaundice. Because a single criterion of PT-INR was employed, the relationship between fat intake and recovery from HCC treatments should be confirmed using other measures in the future.

Although the limited case numbers in this study may have resulted in an inadequate assessment of the biological variability, as neither npRQ nor MHE was associated with body compositions such as BMI, extra cellular water, percent body fat, soft lean mass, or skeletal muscle amount, it is assumed that orally taken fat was directly consumed as an energy source. Nonesterified fatty acid (NEFA) suppresses gluconeogenesis in the liver through insulin secretion. At the same time, however, NEFA desensitizes the liver to insulin *via* insulin receptor substrates, which surpass insulin induction and lead to net elevation of gluconeogenesis [35-40]. Furthermore, fat from diet is absorbed in the form of chylomicrons and is taken up by hepatocytes as a remnant after digestion at the capillary endothelium by lipoprotein lipase [41], promoting gluconeogenesis as a source of energy and substrates such as acetyl-CoA, NADH and ATP. Through β-oxidation processes, acetyl-CoA is subjected to not only gluconeogenesis but also the generation of ketone bodies, which are major energy sources in the brain [42]. In a whole body, npRQ could increase as long as peripheral tissues have glucose and/or ketones to oxidize even under the situation where npRQ decreased in the liver due to gluconeogenesis and ketogenesis [43]. Under PEM, it is teleological for the liver that NEFA uptake is increased in association with up-regulation of gluconeogenesis and ketogenesis [44]. Recently, it was reported that p38 mitogen-activated protein kinase plays a crucial role in the activation of gluconeogenic genes by NEFA [45]. Although the results presented here should be confirmed by a large scale study, our notion is in line with the guideline from the European Society for Clinical Nutrition and Metabolism, which recommends 40% to 50% of non-protein energy requirements (more than 30% of total energy requirements) should be provided by lipid in parenteral nutrition in patients with liver diseases [46]. An appropriate amount of fat intake may have the potential to improve PEM and MHE under the condition such as cirrhosis, in which sugar and protein metabolisms cannot work properly [47,48].

## Conclusions

This study suggested that PEM is a common feature in patients with HCC, and energy state can quickly change based on dietary deviation, which affects various clinical manifestations and recovery from invasive treatments. These findings strongly suggest that nutritional intervention especially for fat intake should be involved in the HCC treatment scheme both at home and in the hospital. Because a hypermetabolic state and inappropriate nutritional usage may hamper the calculation of an exact energy requirement in cirrhotic patients, nutritional supports should be conducted based on a nutritional assessment, which includes nitrogen balance, npRQ and MHE.

## Abbreviations

BCAA: Branched-chain amino acids; BEE: Basal energy expenditure; HCC: Hepatocellular carcinoma; NEFA: Nonesterified fatty acid; npRQ: Non-protein respiratory quotient; NP- test: Neuropsychiatric test; PEM: Protein-energy malnutrition; PT-INR: Prothrombin time; REE: Resting energy expenditure; TUN: Total urea nitrogen; UUN: Urine urea nitrogen; VCO2: Carbon dioxide production; VO2: Oxygen consumption.

## Competing interests

All authors declare that they have no competing interest to disclose.

## Authors’ contributions

TS designed the research, analyzed data and wrote the manuscript. TK, TK, KY, YSK performed npRQ, MHE, body composition and dietary intake analyses. HN and TM evaluated the results of npRQ and nutritional aspect, respectively, and made disciplinary advice. YA made critical revision of the manuscript for important intellectual content. All authors read and approved the final manuscript.

## References

[B1] Bosch FX Ribes J Diaz M Cleries R Primary liver cancer: worldwide incidence and trends Gastroenterology 2004 127 S5 S16 10.1053/j.gastro.2004.09.011 15508102

[B2] Sherman M Hepatocellular carcinoma: epidemiology, surveillance, and diagnosis Semin Liver Dis 2010 30 3 16 10.1055/s-0030-1247128 20175029

[B3] The Cancer of the Liver Italian Program (CLIP) investigators A new prognostic system for hepatocellular carcinoma: a retrospective study of 435 patients Hepatology 1998 28 751 755 9731568 10.1002/hep.510280322

[B4] Kudo M Chung H Osaki Y Prognostic staging system for hepatocellular carcinoma (CLIP score): its value and limitations, and a proposal for a new staging system, the Japan Integrated Staging Score (JIS score) J Gastroenterol 2003 38 207 215 10.1007/s005350300038 12673442

[B5] Lautz HU Selberg O Korber J Burger M Muller MJ Protein-calorie malnutrition in liver cirrhosis Clin Investig 1992 70 478 486 1392415 10.1007/BF00210228

[B6] Moriwaki H Protein-energy malnutrition in liver cirrhosis J Gastroenterol 2002 37 578 579 10.1007/s005350200091 12162420

[B7] Campillo B Richardet JP Scherman E Bories PN Evaluation of nutritional practice in hospitalized cirrhotic patients: results of a prospective study Nutrition 2003 19 515 521 10.1016/S0899-9007(02)01071-7 12781851

[B8] Cabre E Gonzalez Huix F Abad Lacruz A Esteve M Acero D Fernandez Banares F Xiol X Gassull MA Effect of total enteral nutrition on the short-term outcome of severely malnourished cirrhotics. A randomized controlled trial Gastroenterology 1990 98 715 720 2105256 10.1016/0016-5085(90)90293-a

[B9] Kearns PJ Young H Garcia G Blaschke T O'Hanlon G Rinki M Sucher K Gregory P Accelerated improvement of alcoholic liver disease with enteral nutrition Gastroenterology 1992 102 200 205 1727754 10.1016/0016-5085(92)91801-a

[B10] de Ledinghen V Beau P Mannant PR Borderie C Ripault MP Silvain C Beauchant M Early feeding or enteral nutrition in patients with cirrhosis after bleeding from esophageal varices? A randomized controlled study Dig Dis Sci 1997 42 536 541 10.1023/A:1018838808396 9073135

[B11] Muller MJ Malnutrition and hypermetabolism in patients with liver cirrhosis Am J Clin Nutr 2007 85 1167 1168 17490949 10.1093/ajcn/85.5.1167

[B12] Kohn MR Madden S Clarke SD Refeeding in anorexia nervosa: increased safety and efficiency through understanding the pathophysiology of protein calorie malnutrition Curr Opin Pediatr 2011 23 390 394 10.1097/MOP.0b013e3283487591 21670680

[B13] Mehanna H Nankivell PC Moledina J Travis J Refeeding syndrome–awareness, prevention and management Head Neck Oncol 2009 1 4 10.1186/1758-3284-1-4 19284691 PMC2654033

[B14] Savastano DM Covasa M Adaptation to a high-fat diet leads to hyperphagia and diminished sensitivity to cholecystokinin in rats J Nutr 2005 135 1953 1959 16046722 10.1093/jn/135.8.1953

[B15] Legendre A Papakonstantinou E Roy MC Richard D Harris RB Differences in response to corticotropin-releasing factor after short- and long-term consumption of a high-fat diet Am J Physiol Regul Integr Comp Physiol 2007 293 R1076 1085 10.1152/ajpregu.00592.2006 17581834

[B16] Blundell JE Cooling J King NA Differences in postprandial responses to fat and carbohydrate loads in habitual high and low fat consumers (phenotypes) Br J Nutr 2002 88 125 132 12144716 10.1079/BJNBJN2002609

[B17] Woods SC D'Alessio DA Tso P Rushing PA Clegg DJ Benoit SC Gotoh K Liu M Seeley RJ Consumption of a high-fat diet alters the homeostatic regulation of energy balance Physiol Behav 2004 83 573 578 10.1016/j.physbeh.2004.07.026 15621062

[B18] Kim KW Lee DY Jhoo JH Youn JC Suh YJ Jun YH Seo EH Woo JI Diagnostic accuracy of mini-mental status examination and revised hasegawa dementia scale for Alzheimer's disease Dement Geriatr Cogn Disord 2005 19 324 330 10.1159/000084558 15785033

[B19] Kato A Kato M Ishii H Ichimiya Y Suzuki K Kawasaki H Yamamoto SI Kumashiro R Yamamoto K Kawamura N Hayashi N Matsuzaki S Terano A Okita K Watanabe A Development of quantitative neuropsychological tests for diagnosis of subclinical hepatic encephalopathy in liver cirrhosis patients and establishment of diagnostic criteria-multicenter collaborative study in Japanese Hepatol Res 2004 30 71 78 10.1016/j.hepres.2004.07.001 15519270

[B20] Elia M Livesey G Energy expenditure and fuel selection in biological systems: the theory and practice of calculations based on indirect calorimetry and tracer methods World Rev Nutr Diet 1992 70 68 131 1292242 10.1159/000421672

[B21] Tajika M Kato M Mohri H Miwa Y Kato T Ohnishi H Moriwaki H Prognostic value of energy metabolism in patients with viral liver cirrhosis Nutrition 2002 18 229 234 10.1016/S0899-9007(01)00754-7 11882395

[B22] Okuda K Ohtsuki T Obata H Tomimatsu M Okazaki N Hasegawa H Nakajima Y Ohnishi K Natural history of hepatocellular carcinoma and prognosis in relation to treatment. Study of 850 patients Cancer 1985 56 918 928 10.1002/1097-0142(19850815)56:4<918::AID-CNCR2820560437>3.0.CO;2-E 2990661

[B23] Llovet JM Fuster J Bruix J The Barcelona approach: diagnosis, staging, and treatment of hepatocellular carcinoma Liver Transpl 2004 10 S115 120 10.1002/lt.20034 14762851

[B24] Tateishi R Yoshida H Shiina S Imamura H Hasegawa K Teratani T Obi S Sato S Koike Y Fujishima T Makuuchi M Omata M Proposal of a new prognostic model for hepatocellular carcinoma: an analysis of 403 patients Gut 2005 54 419 425 10.1136/gut.2003.035055 15710994 PMC1774402

[B25] Moriwaki H Shiraki M Fukushima H Shimizu M Iwasa J Naiki T Nagaki M Long-term outcome of branched-chain amino acid treatment in patients with liver cirrhosis Hepatol Res 2008 38 S102 S106 19125940 10.1111/j.1872-034X.2008.00434.x

[B26] Kuroda H Ushio A Miyamoto Y Sawara K Oikawa K Kasai K Endo R Takikawa Y Kato A Suzuki K Effects of branched-chain amino acid-enriched nutrient for patients with hepatocellular carcinoma following radiofrequency ablation: a one-year prospective trial J Gastroenterol Hepatol 2010 25 1550 1555 10.1111/j.1440-1746.2010.06306.x 20796154

[B27] Madden AM Morgan MY Resting energy expenditure should be measured in patients with cirrhosis, not predicted Hepatology 1999 30 P655 664 10.1002/hep.510300326 10462371

[B28] Ferenci P Lockwood A Mullen K Tarter R Weissenborn K Blei AT Hepatic encephalopathy–definition, nomenclature, diagnosis, and quantification: final report of the working party at the 11th World Congresses of Gastroenterology, Vienna, 1998 Hepatology 2002 35 716 721 10.1053/jhep.2002.31250 11870389

[B29] Weissenborn K Ennen JC Schomerus H Ruckert N Hecker H Neuropsychological characterization of hepatic encephalopathy J Hepatol 2001 34 768 773 11434627 10.1016/s0168-8278(01)00026-5

[B30] Stewart CA Smith GE Minimal hepatic encephalopathy Nat Clin Pract Gastroenterol Hepatol 2007 4 677 685 18043677 10.1038/ncpgasthep0999

[B31] Michitaka K Tokumoto Y Uesugi K Kisaka Y Hirooka M Konishi I Mashiba T Abe M Hiasa Y Matsuura B Horiike N Shoda T Onji M Neuropsychiatric dysfunction in patients with chronic hepatitis and liver cirrhosis Hepatol Res 2008 38 1069 1075 10.1111/j.1872-034X.2008.00374.x 19000057

[B32] Kato A Tanaka H Kawaguchi T Kanazawa H Iwasa M Sakaida I Moriwaki H Murawaki Y Suzuki K Okita K Nutritional management contributes to improvement in minimal hepatic encephalopathy and quality of life in patients with liver cirrhosis: A preliminary, prospective, open-label study Heptol Res 2013 43 425 458 10.1111/j.1872-034X.2012.01077.x 22994429

[B33] Food and Nutrition Board, Institute of Medicine Dietary reference intakes, for energy, carbohydrate, fiber, fat, fatty acids, cholesterol, protein, and amino acids 2005 Washington D.C: National Academies Press

[B34] Ernst ND Cleeman J Mullis R Sooter Bochenek J Van Horn L The National Cholesterol Education Program: implications for dietetic practitioners from the Adult Treatment Panel recommendations J Am Diet Assoc 1998 88 1401 1408 3183260

[B35] Bevilacqua S Bonadonna R Buzzigoli G Boni C Ciociaro D Maccari F Giorico MA Ferrannini E Acute elevation of free fatty acid levels leads to hepatic insulin resistance in obese subjects Metabolism 1987 36 502 506 10.1016/0026-0495(87)90051-5 3553852

[B36] Boden G Chen X Ruiz J White JV Rossetti L Mechanisms of fatty acid-induced inhibition of glucose uptake J Clin Invest 1994 93 2438 2446 10.1172/JCI117252 8200979 PMC294452

[B37] Lewis GF Vranic M Harley P Giacca A Fatty acids mediate the acute extrahepatic effects of insulin on hepatic glucose production in humans Diabetes 1997 46 1111 1119 10.2337/diabetes.46.7.1111 9200644

[B38] Rebrin K Steil GM Mittelman SD Bergman RN Causal linkage between insulin suppression of lipolysis and suppression of liver glucose output in dogs J Clin Invest 1996 98 741 749 10.1172/JCI118846 8698866 PMC507484

[B39] Sindelar DK Chu CA Rohlie M Neal DW Swift LL Cherrington AD The role of fatty acids in mediating the effects of peripheral insulin on hepatic glucose production in the conscious dog Diabetes 1997 46 187 196 10.2337/diabetes.46.2.187 9000693

[B40] Wiesenthal SR Sandhu H McCall RH Tchipashvili V Yoshii H Polonsky K Shi ZQ Lewis GF Mari A Giacca A Free fatty acids impair hepatic insulin extraction in vivo Diabetes 1999 48 766 774 10.2337/diabetes.48.4.766 10102693

[B41] Beigneux AP Davies BS Gin P Weinstein MM Farber E Qiao X Peale F Bunting S Walzem RL Wong JS Blaner WS Ding ZM Melford K Wongsiriroj N Shu X de Sauvage F Ryan RO Fong LG Bensadoun A Young SG Glycosylphosphatidylinositol-anchored high-density lipoprotein-binding protein 1 plays a critical role in the lipolytic processing of chylomicrons Cell Metab 2007 5 279 291 10.1016/j.cmet.2007.02.002 17403372 PMC1913910

[B42] Rodney R Bell DR Medical physiology: principles for clinical medicine 2009 Riverwoods, IL, USA: Lippincott Williams & Wilkins

[B43] Glass C Hipskind P Tsien C Malin SK Kasumov T Shah SN Kirwan JP Dasarathy S Sarcopenia and a physiologically low respiratory quotient in patients with cirrhosis: a prospective controlled study J Appl Physiol 2013 114 559 565 10.1152/japplphysiol.01042.2012 23288550 PMC3615594

[B44] Muller MJ Hepatic energy and substrate metabolism: a possible metabolic basis for early nutritional support in cirrhotic patients Nutrition 1998 14 30 38 10.1016/S0899-9007(97)00390-0 9437679

[B45] Collins QF Xiong Y Lupo EG Jr Liu HY Cao W p38 Mitogen-activated protein kinase mediates free fatty acid-induced gluconeogenesis in hepatocytes J Biol Chem 2006 281 24336 24344 10.1074/jbc.M602177200 16803882

[B46] Plauth M Cabre E Campillo B Kondrup J Marchesini G Schutz T Shenkin A Wendon J ESPEN Guidelines on Parenteral Nutrition: hepatology Clin Nutr 2009 28 436 444 10.1016/j.clnu.2009.04.019 19520466

[B47] Yamanaka Okumura H Nakamura T Takeuchi H Miyake H Katayama T Arai H Taketani Y Fujii M Shimada M Takeda E Effect of late evening snack with rice ball on energy metabolism in liver cirrhosis Eur J Clin Nutr 2006 60 067 1072 10.1038/sj.ejcn.1602420 16508643

[B48] Kawaguchi T Itou M Taniguchi E Sakata M Abe M Koga H Oriishi T Imamura Y Kato T Yamada K Sata M Serum level of free fatty acids is associated with nocturnal hypoglycemia in cirrhotic patients with HCV infection: a pilot study Hepatogastroenterology 2011 58 103 108 21510295

